# Surfactant-assisted tungsten oxide for enhanced acetone sensing and solar-driven photocatalysis: exploring the role of surfactants

**DOI:** 10.1039/d5ra02593a

**Published:** 2025-08-05

**Authors:** Abhijeet P. Patil, Suraj S. Patil, Mohaseen S. Tamboli, Shubhangi R. Damkale, Digambar Y. Nadargi, Jyoti D. Nadargi, Imtiaz S. Mulla, Sharad S. Suryavanshi

**Affiliations:** a School of Physical Sciences, Punyashlok Ahilyadevi Holkar Solapur University Solapur-413255 Maharashtra India sssuryavanshi@rediffmail.com; b Department of Physics, Yashavantrao Chavan Institute of Science Satara-415001 India; c Korea Institute of Energy Technology (KENTECH) 21 KENTECH-gil Naju Jeollanam-do 58330 Republic of Korea tamboli.mohseen@gmail.com; d Centre for Materials for Electronics Technology (C-MET) Off Pashan Road, Panchawati Pune-411008 Maharashtra India; e Centre for Materials for Electronics Technology (C-MET) Thrissur-680581 Kerala India digambar_nadargi@yahoo.co.in; f Department of Physics, Santosh Bhimrao Patil College Mandrup Solapur-413221 India; g Former Emeritus Scientist, (C-MET), National Chemical Laboratory Pune-411008 India

## Abstract

Surfactants play a pivotal role in the kinetics of nucleation and accretion of nanoparticles in such a way that they serve as a template for the development of nanostructures, consequently influencing the morphology, dimensions, and other surface properties. Herein, we report the influence of cationic and anionic surfactants (CTAB, SDS, PVP and HMT) on the development of pristine WO_3_ nanostructures and their impact on gas sensing and photocatalytic properties of WO_3_. The various surfactant-assisted WO_3_ nanostructures were synthesized *via* a straightforward hydrothermal route and systematically analyzed using XRD, FESEM-EDAX, TEM/HRTEM, XPS, UV-Vis, and BET measurements. Gas sensing properties of various oxidizing and reducing gases revealed superior selectivity towards acetone. Among the various surfactant-assisted WO_3_, CTAB/WO_3_ exhibited an excellent response of 84.84% towards 100 ppm acetone at an optimal operating temperature of 300 °C. The CTAB/WO_3_ sensor exhibited a linear response to acetone at lower concentrations, showing a 4.8% response at 0.8 ppm, which delineates the threshold between healthy and diabetic breath acetone levels. At 1.8 ppm, the sensor recorded 8.1% response, aligning with diabetes values reported by National Institute for Occupational Safety and Health (NIOSH). Moreover, photocatalytic performance evaluations demonstrated a methylene blue degradation efficiency of 47.19% under natural solar irradiation. This work will motivate researchers in developing high performance acetone gas sensors and photocatalytic dye-degradation by the integration of appropriate surfactants in WO_3_ nanostructures.

## Abstract 2

Schematic illustration of theme of the reported work.

## Graphical abstract

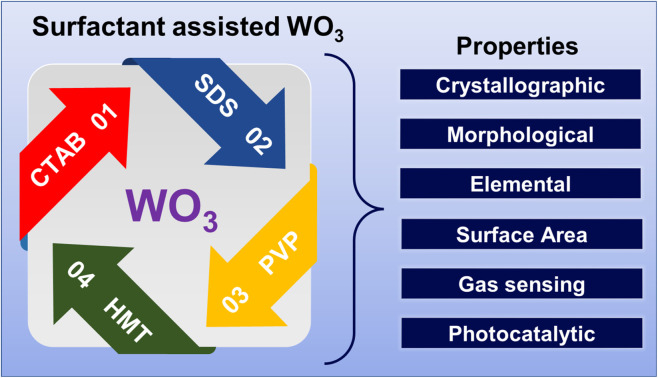

## 1. Introduction

Surfactants are among the most versatile compounds, with a wide spectrum of applications in the chemical industry, pharmaceuticals, petroleum and high-technology fields such as electronic printing, magnetic recording, biotechnology, micro-electronics, sensors and many more.^1,2^ A surfactant, an abbreviation of “surface-active agent,” is a substance that, when present in a system at a low concentration, has the ability to adsorb onto the surface or interface of the system and significantly alter its free energy.^3^ It serves as a template for the development of nanostructures, thereby regulating the morphology, dimensions and other surface characteristics. Moreover, it helps to prevent the aggregation of nanostructures during the formation stage.^4^ The nature of the surfactant molecule directly influences the formation of nanostructures which depends upon surfactant parameters like charge, functional group, surfactant shape, hydrophobic/hydrophilic tail length, surfactant concentration, and solution pH. When surfactants are involved in chemical reactions, especially in the development of metal oxides, they play an important role in tuning the surface area and thereby properties of the parent metal oxide.^5–7^

With this motivation, in the present work, a focus is made to enhance the properties of metal oxide (WO_3_ in the present case) with an emphasis on role of different surfactants (cationic, anionic and both) on the development of metal oxide and thereby its dual application, as gas sensor and photocatalytic dye-degradation in the natural sunlight.^8^ Nonetheless, as per our knowledge, minimal to no research is reported on dual application (gas sensing and photocatalysis) of WO_3_ nanostructures using different surfactants in a single tool box, which can certainly broaden the related research.

Particularly selecting the metal oxide as WO_3_ in the present investigations is due to its exceptional chemical stability, superior electrochemical performance, high electron mobility, significant photoactivity, multiple crystalline polymorphs, straightforward synthesis, n-type conductivity, a broad band gap (∼3 eV) and notable photosensitivity.^9^ The inherent conductivity of WO_3_ arises from its nonstoichiometric composition, leading to oxygen vacancy defects within the lattice, leading to ideal property for gas sensing and photocatalytic characteristics to be studied.

The specific choice of the surfactant is made by keeping in view of their surface charges; for example – (i) cetyltrimethylammonium bromide (CTAB) is a cationic structure directing surfactant that promotes anisotropic growth *via* micelle templating and mesoporous, (ii) sodium dodecyl sulphate (SDS), an anionic surfactant, stabilizes hydrolyzed species through electrostatic interactions, (iii) polyvinylpyrrolidone (PVP) as an additional anionic surfactant, which acts as a capping agent, preventing agglomeration by sterically stabilizing nuclei, and (iv) hexamethylenetetramine (HMT) as a weak base, regulates pH by releasing ammonia to control the formation of nanostructures.^10–12^

**Fig. 1:**
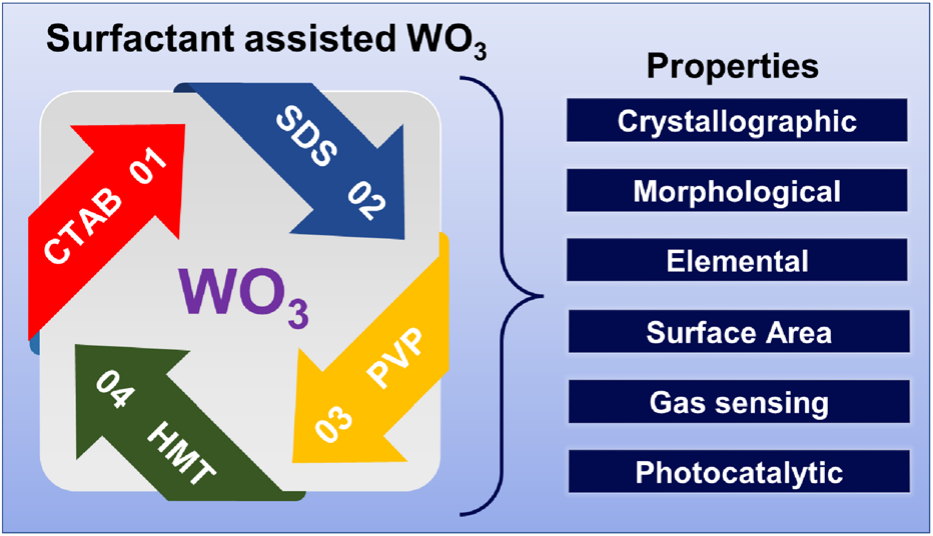
Schematic illustration of theme of the reported work.

Fig. 1 highlights the surfactant-assisted synthesis of WO_3_ using CTAB, SDS, PVP and HMT. Its impact on crystallographic, morphological, elemental, band gap, surface area, gas sensing, and photocatalytic properties were studied.

## 2. Experimental section

**Fig. 2:**
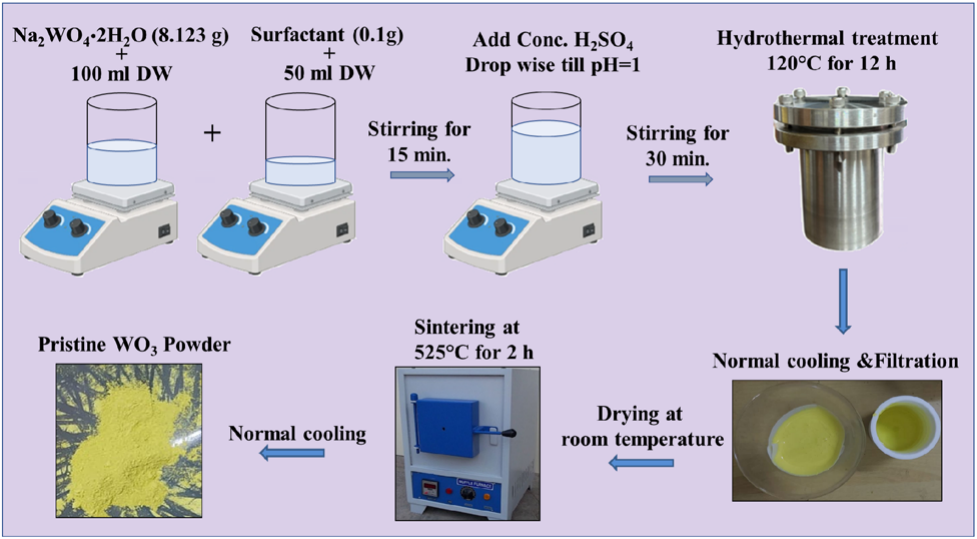
Synthetic protocols of surfactant assisted-WO_3_.

To synthesize WO_3_ nanostructures, precursors such as sodium tungstate dihydrate and sulfuric acid (purchased from Sigma Aldrich) were used without further purification, along with different surfactants including CTAB (cetyltrimethylammonium bromide), SDS (sodium dodecyl sulfate), PVP (polyvinylpyrrolidone), and HMT (hexadecyltrimethylammonium bromide). WO_3_ nanostructure with different surfactants was prepared by a facile one-step hydrothermal process. In a beaker, 8.12 g of sodium tungstate dihydrate (Na_2_WO_4_·2H_2_O) was added to 100 mL of distilled water and stirred for 15 min until complete dissolution was occurred. Another beaker containing 0.1 g of CTAB was added to 50 mL of distilled water and stirred to dissolve completely. The solutions in the two beakers were combined together and stirred again for 5 min. To the resulting solution, concentrated H_2_SO_4_ was added dropwise upon stirring, until the pH reached 1. The final obtained solution was stirred for 30 min and transferred to a Teflon liner for hydrothermal treatment in a reaction temperature of 120 °C for 12 h. After filtration, a lemon-yellow-colored precipitate was obtained, which was washed with ample amount of distilled water and then dried at 60 °C overnight. The WO_3_ powders were further annealed at 525 °C for 2 h. A similar procedure was followed for the synthesis of WO_3_ with other surfactants such as SDS, PVP and HMT. The schematic representation of synthetic protocol of surfactant-assisted WO_3_ is shown in Fig. 2. The details on the characterization techniques used are provided in the SI (SI-I).

## 3. Results and discussion

**Fig. 3:**
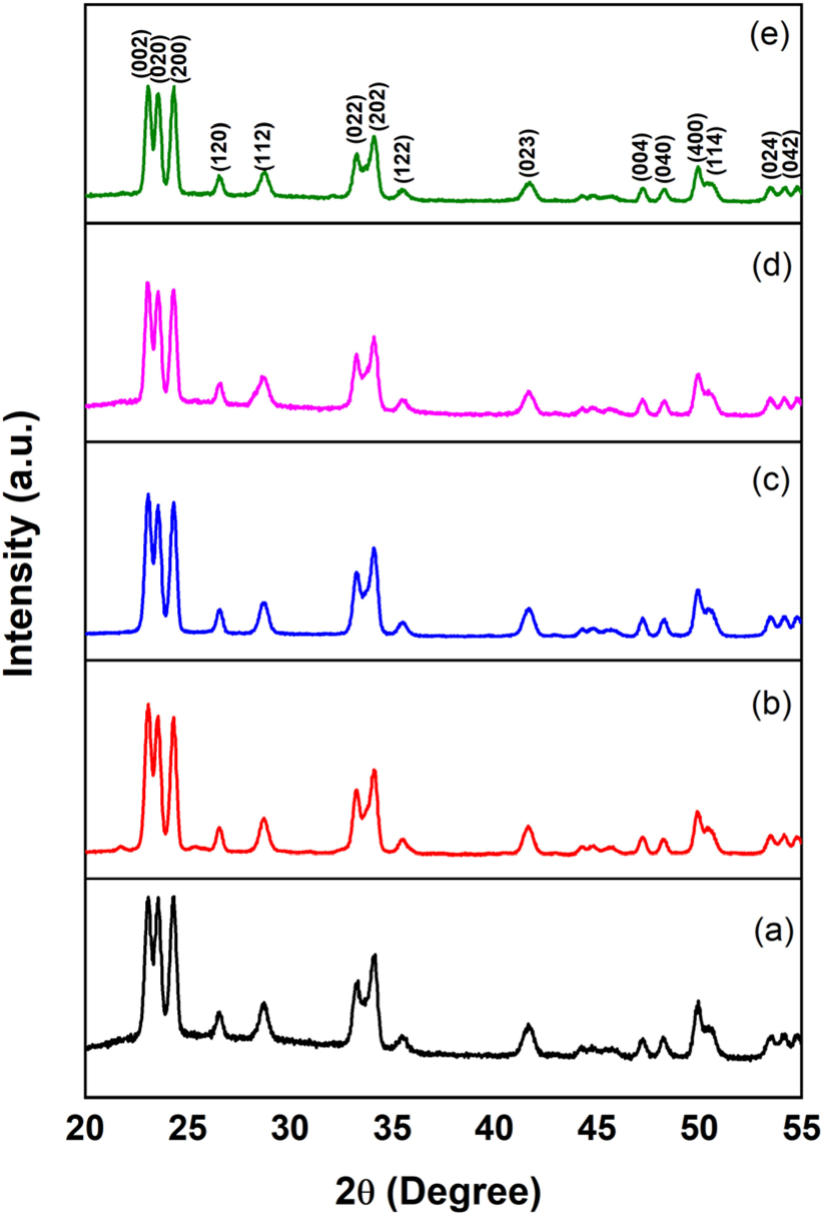
XRD patterns of (a) pristine WO_3_ (b) CTAB–WO_3_ (c) SDS–WO_3_ (d) PVP–WO_3_ (e) HMT–WO_3_.

The phase purity and crystallographic structure of hydrothermal synthesized pristine WO_3_ and various surfactant-assisted WO_3_ (CTAB, SDS, PVP and HMT) samples were confirmed by X-ray diffraction analysis. The diffraction peaks observed at 2*θ* values of 23.11°, 23.59°, 24.35°, 26.58°, 28.76°, 33.20°, 34.16°, 35.58°, 42.62°, 47.25°, 48.25°, 49.93°, and 50.72° are indexed to the (002), (020), (200), (120), (112), (022), (202), (122), (023), (004), (040), (400) and (144) crystal planes, respectively as shown in Fig. 3. These results are in good agreement with the standard ICDD card number 83-0950, confirming the formation of a monoclinic crystal structure. The peak intensities of surfactant-assisted WO_3_ samples are slightly lower than those of pristine WO_3_, indicating changes in crystallinity while maintaining phase purity, as no additional peaks were observed. The crystallite size was calculated using the Debye–Scherrer equation.^13^ The increasing trend in the crystallite size for PVP, SDS and HMT-assisted WO_3_ can be primarily attributed to the weaker interaction with WO_4_^2−^ precursor ions during the synthesis process. In contrast, the CTAB-assisted WO_3_ samples showed smallest crystallite size (21.56 nm, Table 1) amongst all the samples. The reduced crystallite size enhances the surface-to-volume ratio, facilitating better adsorption of active oxygen species on the surface.^14–16^ The crystallite size of WO_3_ decreases after adding CTAB during hydrothermal synthesis because CTAB works as a structural, capping and stabilizing agent. It controls the growth of particles and stops them from becoming too large, resulting in smaller and more uniform WO_3_ nanoparticles. Also CTAB–WO_3_ show highest dislocation density and microstrain, suggesting a significant presence of lattice defects and distortions during the synthesis process. These structural imperfections facilitate charge carrier separation, hence diminishing recombination and consequently augmenting light absorption. This might enhance photocatalytic efficacy.^17^

**Table 1:** Crystallite size, lattice parameter, dislocation density, and micro strain for WO_3_ samples with different surfactants

Sample id	Crystallite size (nm)	Lattice parameters	Dislocation density (*ρ*) 10^16^ (lines per m^2^)	Microstrain (*ε*) 10^−2^ (lines per m^4^)
Pristine WO_3_	22.40	*a* = 7.30 Å	0.19	0.74
		*b* = 7.53 Å		
		*c* = 7.68 Å		
		*β* = 90.89°		
CTAB–WO_3_	21.56	*a* = 7.30 Å	0.21	0.78
		*b* = 7.53 Å		
		*c* = 7.68 Å		
		*β* = 90.89°		
SDS–WO_3_	22.27	*a* = 7.30 Å	0.20	0.75
		*b* = 7.53 Å		
		*c* = 7.68 Å		
		*β* = 90.89°		
PVP–WO_3_	23.64	*a* = 7.30 Å	0.17	0.72
		*b* = 7.53 Å		
		*c* = 7.68 Å		
		*β* = 90.89°		
HMT–WO_3_	23.25	*a* = 7.30 Å	0.18	0.73
		*b* = 7.53 Å		
		*c* = 7.68 Å		
		*β* = 90.89°		

The average crystallite size (*D*) was calculated by Debye–Scherrer formula

1 *D* = *kλ*/(*β* cos(*θ*))

where, *k* is Scherrer constant, *λ* is X-ray wavelength, *β* is Full Width at Half Maximum (FWHM) of the diffraction peak and *θ* is Bragg angle.

Dislocation density (*δ*) was calculated using the formula

2 *δ* = 1/*D*^2^

where, *D* is crystallite size.

Microstrain (*ε*) was calculated using the formula

3 *ε* = *β*/4 tan *θ*

where, *β* is the Full Width at Half Maximum (FWHM) of the diffraction peak and *θ* is Bragg angle.

**Fig. 4:**
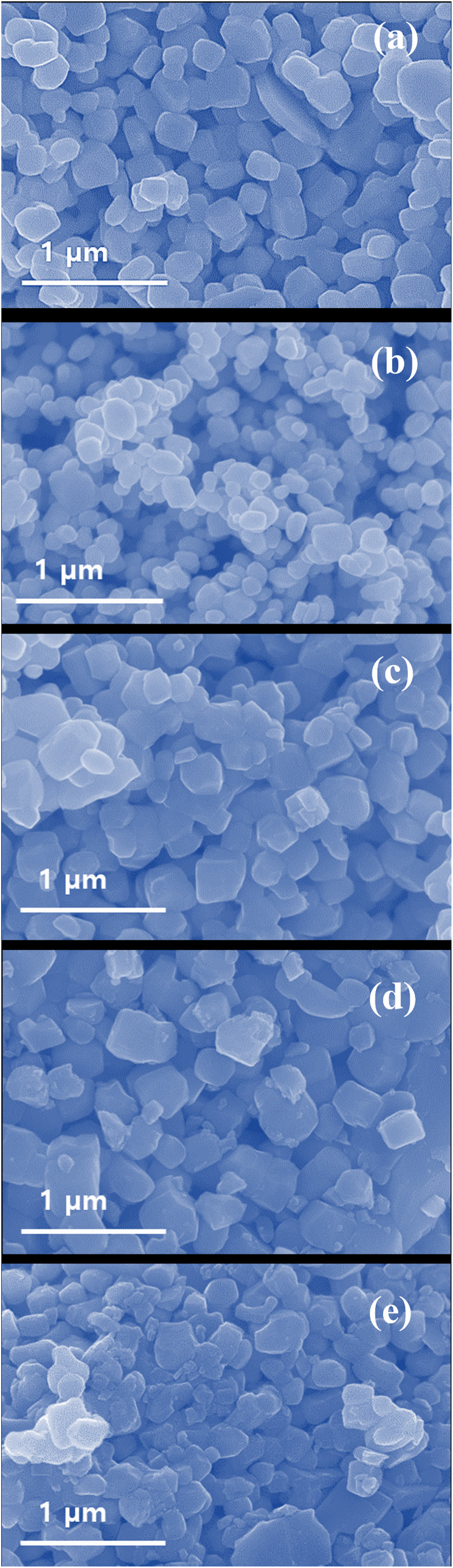
FESEM images of samples (a) pristine WO_3_, (b) CTAB–WO_3_, (c) SDS–WO_3_, (d) PVP–WO_3_, and (e) HMT–WO_3_.

The Field Emission Scanning Electron Microscopy (FESEM) images of pristine WO_3_ and surfactant-assisted WO_3_ samples are shown in Fig. 4a–e. This clearly shows the different surfactants play different roll in tuning the properties of the parent material. The pristine WO_3_ (Fig. 4a) shows a rectangular nanoplate-like morphology with a uniform average particle size of 226 nm. The cationic surfactant as a capping agent CTAB-assisted WO_3_ (Fig. 4b) sample shows a smaller nanoplate-like structure with average particle size of 156 nm. Fig. 4c depicts an anionic surfactant SDS-assisted WO_3_ exhibits nanoplate morphology, with relatively uniform particles having an average size of 188 nm. Furthermore, PVP-assisted WO_3_ (Fig. 4d) samples show slightly larger nanoplate structures with an average size of 215 nm. Moreover, HMT-assisted WO_3_ (Fig. 4e) displays quite distorted nano-plates with an irregular structure and an average particle size of 142 nm. The reduced particle size particularly in the CTAB assisted WO_3_ sample could be attributed to the surface capping effect of CTAB, which inhibits excessive crystal growth and helps in aggregation leading to the formation of smaller and more uniform particles.^18^

**Fig. 5:**
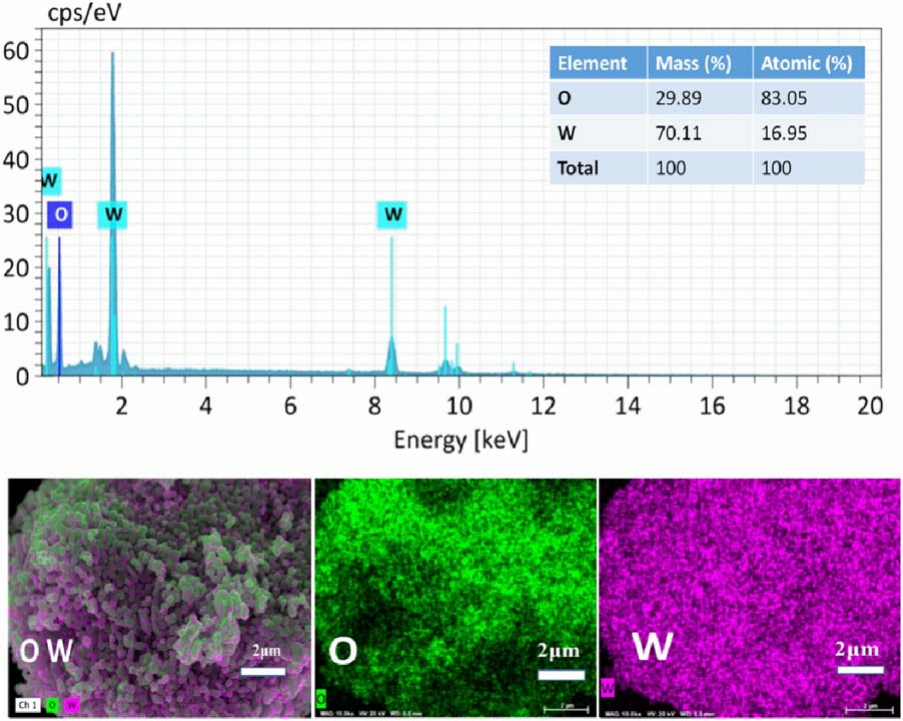
EDS analysis and elemental mapping of the optimized CTAB–WO_3_ sample.

The EDS analysis and elemental mapping of CTAB–WO_3_ sample is shown in Fig. 5, which confirm its composition as (i) atomic%: 83.05% (W) and 16.95% (O), (ii) mass%: 70.11%, (W) and 29.89% (O). This composition corresponds to the expected WO_3_ structure, with no additional impurities which demonstrates the high purity of the synthesized stoichiometric WO_3_ material. Moreover, the elemental mapping images clearly show the uniform and homogeneous distribution of W and O throughout the sample, further validating the absence of impurities or phase segregation.

**Fig. 6:**
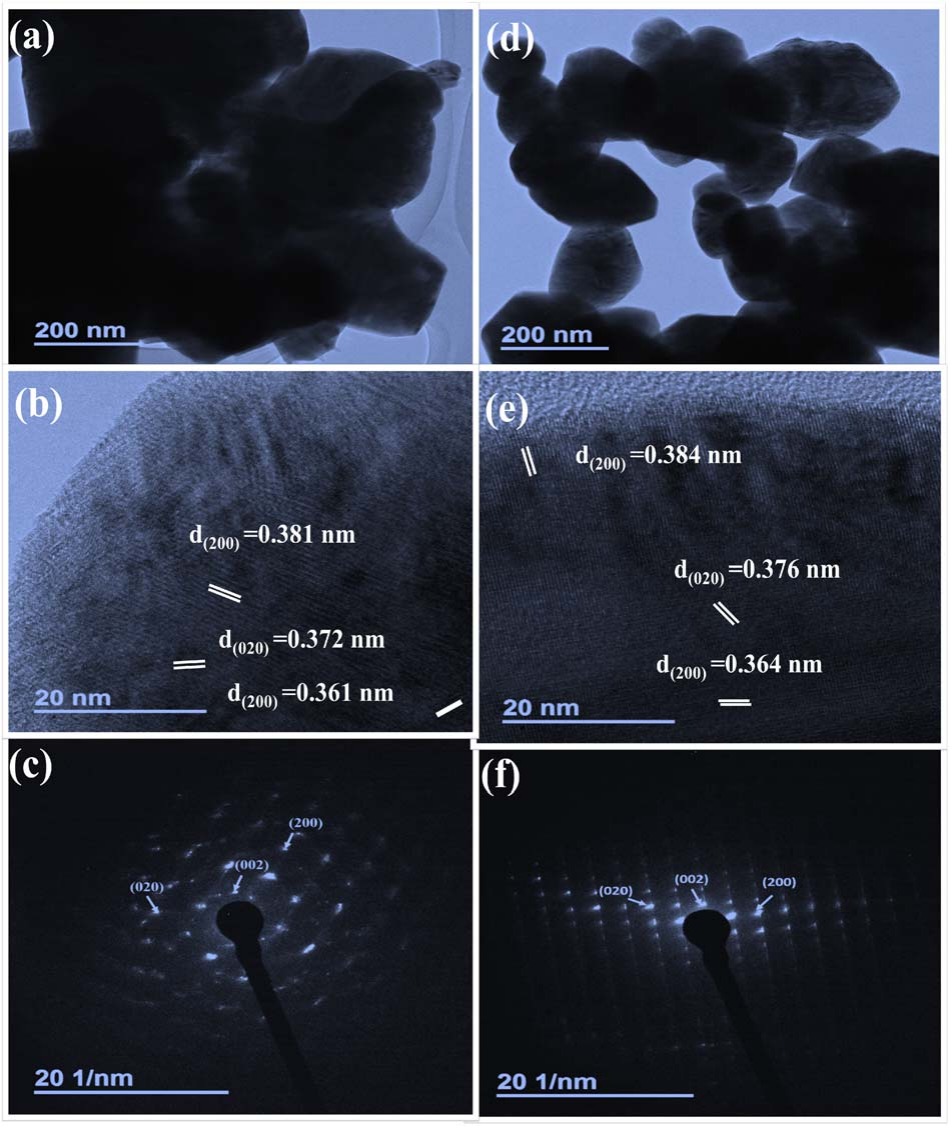
TEM, HRTEM and SAED analysis of pristine WO_3_ (a–c) and CTAB–WO_3_ (d–f), displaying morphological and structural characteristics with indexed SAED patterns.

Fig. 6 showcase the TEM, HRTEM, and SAED pattern of pristine WO_3_ (a, b and c) and CTAB–WO_3_ (d, e and f), respectively. TEM images (Fig. 6a and d) reveal the distinct size variation in the WO_3_ nanoplates. Moreover, the porous network structure is seen in case of CTAB assisted WO_3_. Pristine WO_3_ (Fig. 6a) exhibits agglomerated nanoplates with an average particle size of 230 nm. HRTEM (Fig. 6b) shows lattice fringes with interplanar spacing of 0.381 nm, 0.372 nm, and 0.361 nm, corresponding to the (200), (020) and (200) planes of monoclinic WO_3_, indicating high crystallinity. The SAED pattern (Fig. 6c and f) confirm its crystalline nature, revalidating the XRD analysis. Similarly, CTAB-assisted WO_3_ (Fig. 6d) forms uniform nanoplates with an average particle size of 160 nm due to cationic capping agent surfactant that promotes anisotropic growth of nanoparticles. HRTEM (Fig. 6e) shows increased interplanar spacing (0.384 nm, 0.376 nm, 0.364 nm), corresponding to the (200), (020) and (200) planes of monoclinic WO_3_, suggesting reduced crystallite size, higher porosity, and enhanced surface area. These changes result in a larger number of active sites, assisting more gas adsorption and charge transport, thereby significantly enhancing gas sensing and photocatalytic performance.^19,20^

**Fig. 7:**
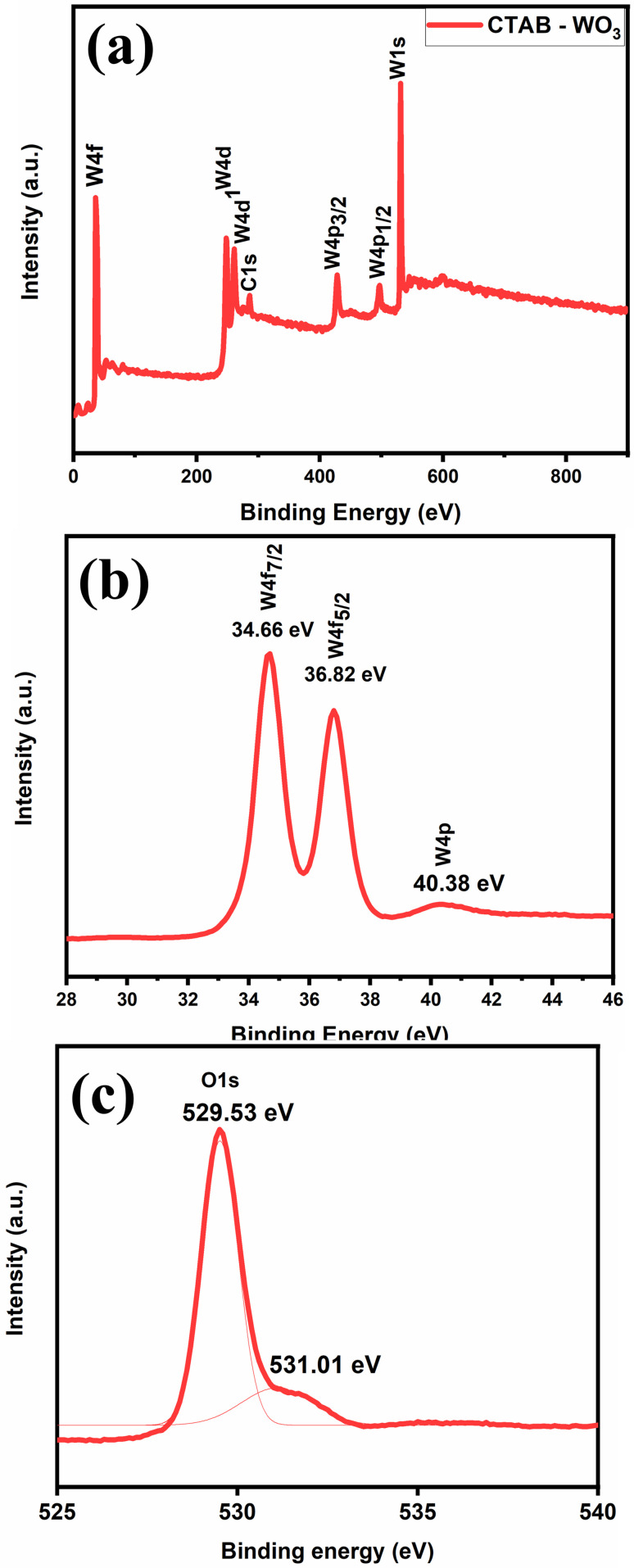
(a) The XPS survey spectrum provides an overview of the sample's elemental composition, highlighting the presence of various elements. (b) The XPS spectra of the tungsten (W) region show the spin–orbit split peaks of W 4f and W 4p, which are crucial for understanding the chemical state of tungsten in the sample. (c) The O 1s spectrum is depicted with detailed spectral separation, offering insights into the oxygen bonding environment and its interactions within the sample.

X-ray photoelectron spectroscopy (XPS) was employed to determine the oxidation state and surface chemical composition of CTAB–WO_3_ (Fig. 7). In Fig. 7a, the spectrum reveals multiple peaks corresponding to various binding energies, effectively identifying the elemental composition and oxidation states present in the sample. Notable peaks corresponding to W 4f, W 4d, W 4p and O 1s, confirm the presence of WO_3_ in the material. Fig. 7b displays the high-resolution W 4f spectrum, revealing distinct doublet peaks at binding energies of 34.66 eV (W 4f_7/2_) and 36.82 eV (W 4f_5/2_), which are characteristic of tungsten in the W^6+^ oxidation state. These peaks indicate that tungsten is bonded oxygen within the WO_3_ structure, confirming the complete oxidation as expected. Additionally, a minor peak at 40.38 eV (W 4p) corresponds to another tungsten orbital, further substantiating the presence of tungsten in its oxidized form. The O 1s spectrum is as shown in Fig. 7c of CTAB–WO_3_ shows two components: a peak at 529.53 eV (lattice oxygen) and a second peak at 531.01 eV attributed to oxygen vacancies and surface hydroxyls. CTAB–WO_3_ shows enhanced oxygen vacancy formation due to surfactant-induced control over crystal growth and surface defects, which increases active sites for gas adsorption, facilitates charge transfer, and significantly boosts gas sensing performance.^21^

Fig. 7c presents the high-resolution O 1s spectrum of WO_3_, exhibits two distinct peaks corresponding to different oxygen environments. The characteristic peak at 529.53 eV corresponds to lattice oxygen (O^2−^), confirming a well-formed tungsten oxide with oxygen atoms strongly bonded to tungsten within the bulk structure. On the other hand, the peak at 531.01 eV is attributed to surface oxygen species or oxygen vacancies, suggesting the presence of hydroxyl groups or adsorbed oxygen and defects (vacancies) which play a crucial role to enhance acetone sensing performance^16^

**Fig. 8:**
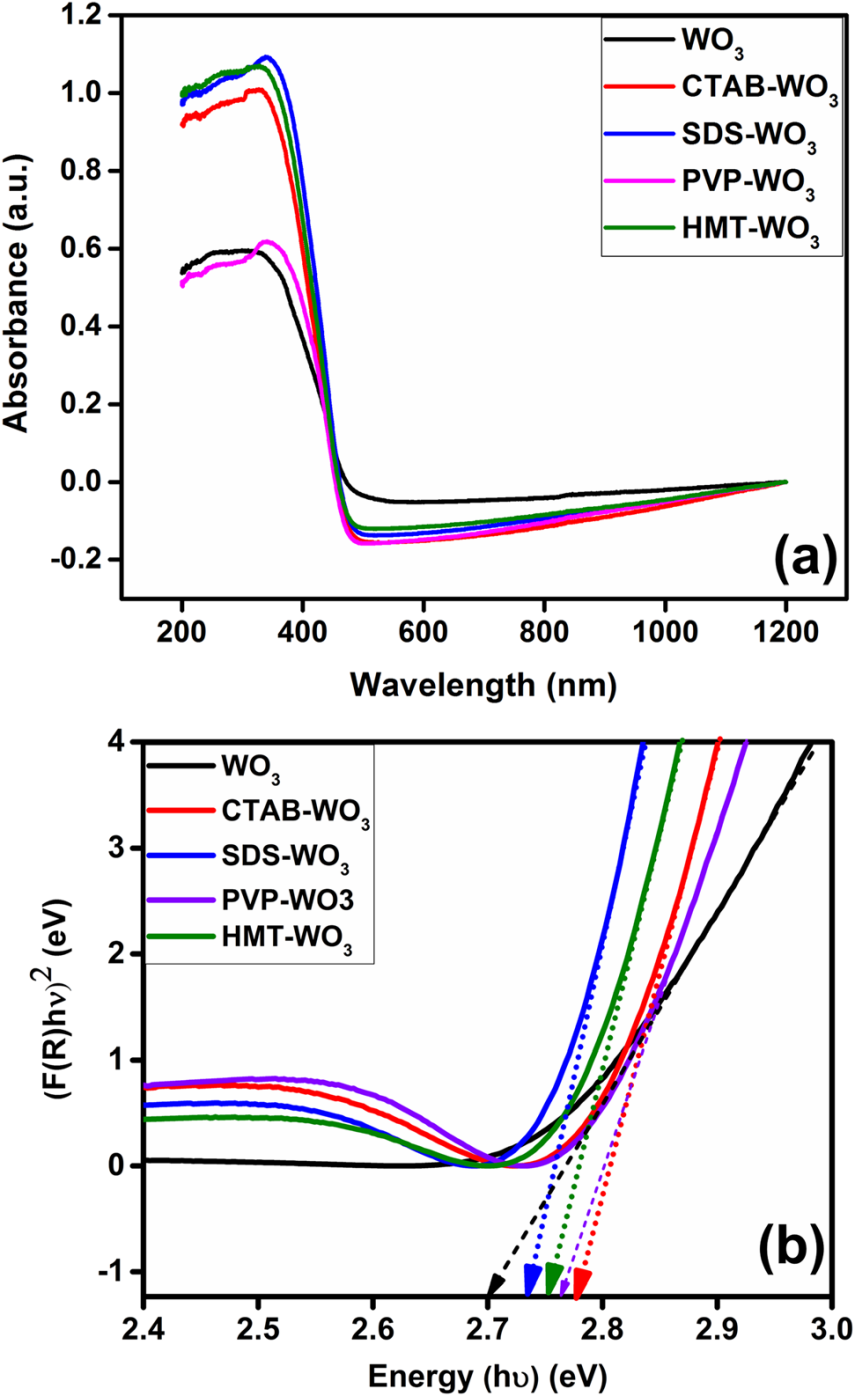
(a) UV-Vis-NIR absorbance spectra, and (b) Tauc plots of pristine WO_3_ and its composites with CTAB, SDS, PVP, and HMT, showing the determination of optical band gap energies (*E*_g_).

The UV-Vis-NIR absorbance spectra of pristine WO_3_ and surfactant-assisted WO_3_ samples (CTAB–WO_3_, SDS–WO_3_, PVP–WO_3_ and HMT–WO_3_) are presented in Fig. 8a. All samples exhibit strong absorbance in the UV region with a distinct absorption edge between 350–450 nm, characteristic of semiconductor behaviour. These absorbance spectra serve as the foundational data for constructing the Tauc plots used to estimate the optical band gap energies. The observed shifts in the absorption edge among different surfactant-assisted WO_3_ samples suggest slight variations in band gap. The Tauc plots (Fig. 8b) demonstrating the relationship between (*F*(*R*)*hν*)^2^ and photon energy (*hν*) for various tungsten trioxide (WO_3_) samples, synthesized without and with different surfactants, was used to determine the band gap energy (*E*_g_) of the materials. The calculated band gap energies of all the samples are WO_3_ (2.77 eV), CTAB–WO_3_ (2.69 eV), SDS–WO_3_ (2.73 eV), PVP–WO_3_ (2.76 eV) and HMT–WO_3_ (2.75 eV). Among all the samples, CTAB–WO_3_ (2.69 eV) is considered as the optimal gas sensor sample due to their slightly narrower band gap as compared to the other samples, as shown in Fig. 8. This band gap of WO_3_ is favourable for gas sensing and photocatalytic application.

**Fig. 9:**
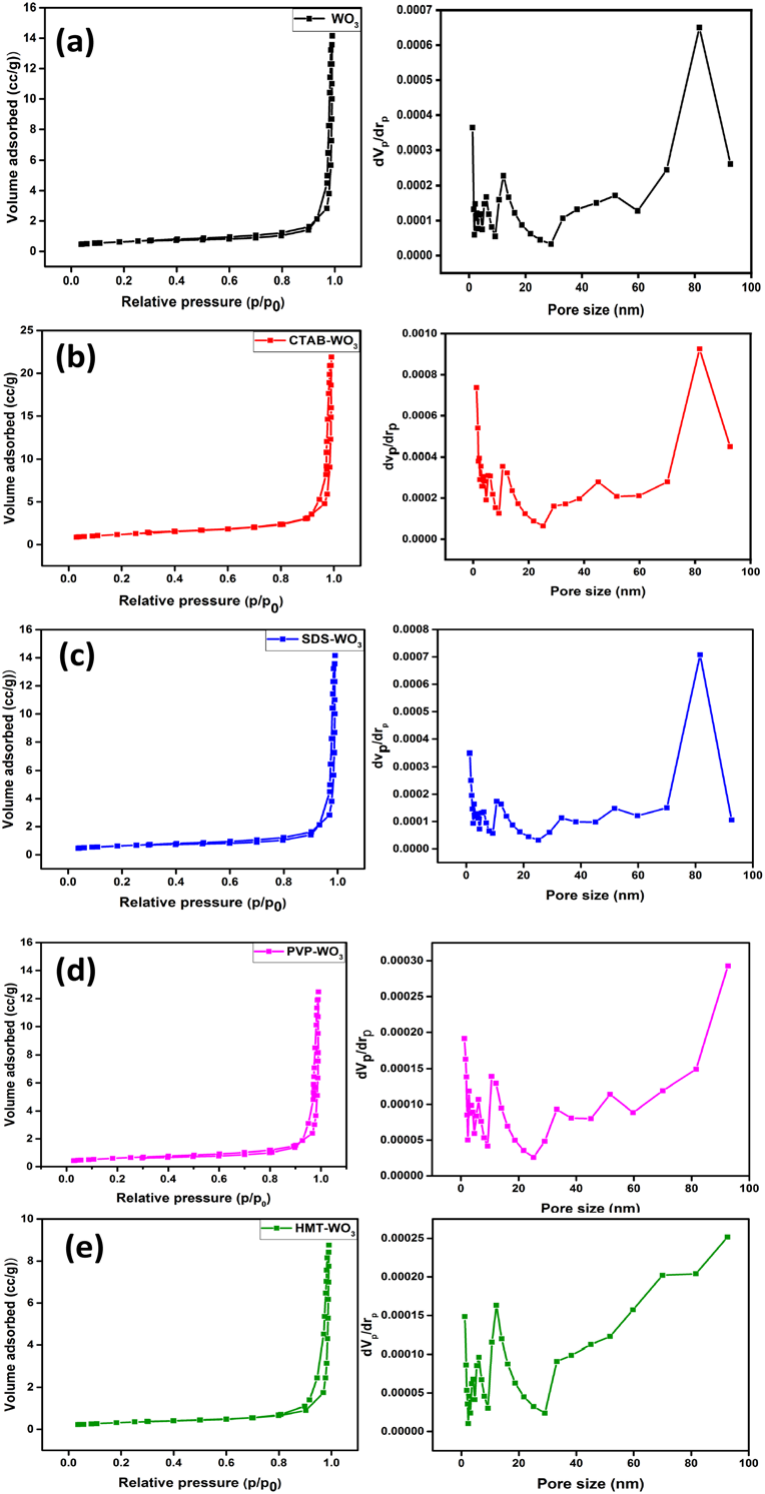
Nitrogen adsorption–desorption isotherms and pore size distribution of (a) pristine WO_3_ (b) CTAB–WO_3_ (c) SDS–WO_3_ (d) PVP–WO_3_ (e) HMT–WO_3_.

Fig. 9 presents the nitrogen adsorption–desorption isotherms of pristine and surfactant-assisted WO_3_ samples, clearly indicating variations in surface area and porosity. All samples exhibit type IV isotherms with H3-type hysteresis loops, characteristic of mesoporous materials with slit-like pores. The observed hysteresis confirms the presence of mesopores with diameters ranging from 2 to 50 nm, where capillary condensation occurs.^22^ Among them, CTAB–WO_3_ shows the highest surface area (4.22 m^2^ g^−1^), moderate pore size (31.39 nm), and good pore volume (0.033 cm^3^ g^−1^). This porous structure provides more active sites for gas adsorption and better pathways for gas diffusion, which directly enhances the sensing response. The balanced pore size allows effective gas transport, and the high surface area increases interaction with acetone molecules. SDS–WO_3_ shows a surface area of 2.18 m^2^ g^−1^, pore diameter of 34.54 nm, and pore volume of 0.019 cm^3^ g^−1^, which is comparable to pristine WO_3_ but lower than CTAB–WO_3_. While SDS helps maintain mesoporosity, the relatively lower surface area and pore volume. In contrast, PVP–WO_3_, though it has the highest pore volume (0.110 cm^3^ g^−1^), shows a lower surface area (1.49 m^2^ g^−1^) and slightly smaller pores (29.98 nm), which may limit the number of active adsorption sites. HMT–WO_3_ has the lowest surface area (0.99 m^2^ g^−1^) and pore volume (0.013 cm^3^ g^−1^), with a large pore diameter (53.55 nm). Thus, CTAB–WO_3_ offers the best combination of surface area, pore size, and pore volume, as summarized in Table 2, making it the most effective material for high-performance gas sensors in this study. These results underline the crucial role of surfactant selection in tuning the microstructure and enhancing gas sensing and photocatalytic behaviour of WO_3_-based materials.

**Table 2:** BET surface area, pore size, and pore volume distribution of pristine and surfactant assisted WO_3_

Sample id	Surface area (m^2^ g^−1^)	Pore diameter (nm)	Pore volume (cm^3^ g^−1^)
WO_3_	2.26	38.14	0.021
CTAB–WO_3_	4.22	31.39	0.033
SDS–WO_3_	2.18	34.54	0.019
PVP–WO_3_	1.49	29.98	0.110
HMT–WO_3_	0.99	53.55	0.013

### 3.1 Gas sensing application

The gas sensing performance of as developed WO_3_ was evaluated for reducing gases: acetone (CH_3_COCH_3_), methanol (CH_3_OH), ethanol (C_2_H_5_OH), propanol (C_3_H_8_OH), formaldehyde (CH_2_O), and ammonia (NH_3_), as well as oxidizing gas (NO_*x*_). The measurements were conducted at operating temperatures ranging from 200 °C to 400 °C under consistent experimental conditions. The sample's resistance in both air and target gas environments was carefully analysed to assess its sensitivity and selectivity. Additionally, the sensor's reliability were also confirmed through repeatability and reproducibility studies, ensuring its suitability for useful applications.

The sensor response in case of reducing gas (*S*%) was measured as

4 *S* (%) = (*R*_a_ − *R*_g_)/*R*_a_ × 100

where *R*_a_ and *R*_g_ represent the sensor's resistance in air and test gas.

While, for oxidizing gas, sensor response (*S*%) was measured as

5 *S* (%) = (*R*_g_ − *R*_a_)/*R*_g_ × 100

where, *R*_a_ and *R*_g_ represent the sensor's resistance in air and test gas.

#### 3.1.1 Gas sensing mechanism

WO_3_ is an n-type semiconducting material, where majority charge carriers are electrons. When WO_3_ comes in contact with air, the oxygen molecules in the air will get adsorbed onto the surface of WO_3_ and by capturing the free electrons of the conduction band, adsorbed oxygen molecules change into O_2_^−^, O^−^ or O^2−^, which is known as chemisorbed oxygen.

6 O_2_(g) + e^−^ ⇔ 2O^−^(ad)

7 O_2_^−^(ad) + e^−^ ⇔ 2O^−^(ad)

8 2O(g) + e^−^ ⇔ 2O^−^(ad)

**Fig. 10:**
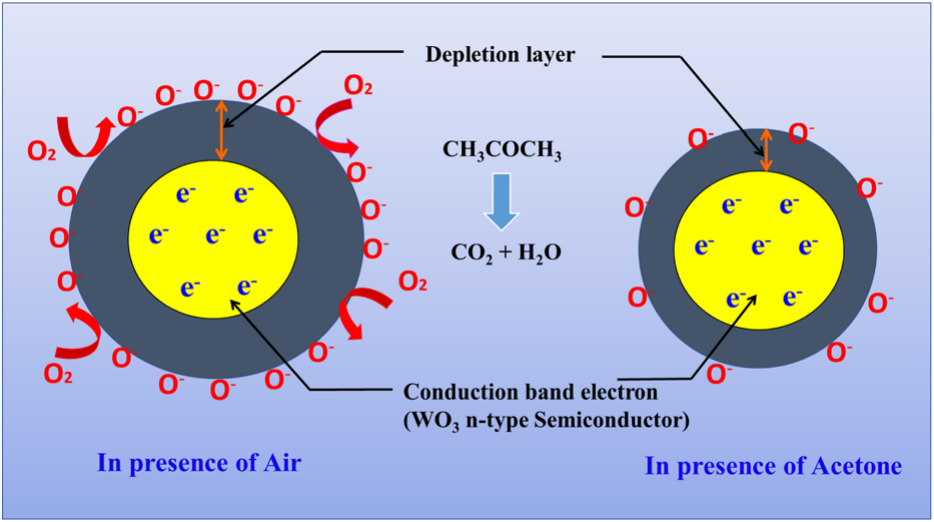
Schematic representation of acetone gas sensing mechanism.

This layer of chemisorbed molecule results in the formation of an electron depletion layer on the surface of WO_3_ as shown in Fig. 10. Upon exposure to reducing gases, the width of the depletion layer decreases, because these gases react with chemisorbed oxygen to release the captured electrons. Similarly, when these materials are exposed to oxidizing gases, the width of the depletion layer increases by capturing more electrons from the conduction band.

#### Reaction with acetone

When acetone vapors come into contact with the WO_3_ sensor, they react with the adsorbed ionic oxygen species:

9 CH_3_COCH_3_(gas) + 6O^−^ → 3CO_2_(g) + 6H_2_O(g) + e^−^

In surfactant-assisted WO_3_ materials, more adsorbed oxygen species will be observed due to an increased surface-to-volume ratio as confirmed by BET results, which causes easy reactiveness with acetone vapour.^23^

In our study, the gas sensing measurements were carried out under normal laboratory conditions with ambient pressure 93 kPa and relative humidity of around 80%. However, it is understood that environmental factors like humidity and pressure can strongly affect sensor performance, especially in real-life applications like breath analysis for diabetes, where humidity is naturally high. Humidity can compete with acetone molecules for adsorption on the sensor surface and may change the resistance and response values.

Further details on the fabrication process and sensor setup, along with the schematic diagram of the gas sensing unit, are provided in the SI (SI-II).

#### 3.1.2 Selectivity

**Fig. 11:**
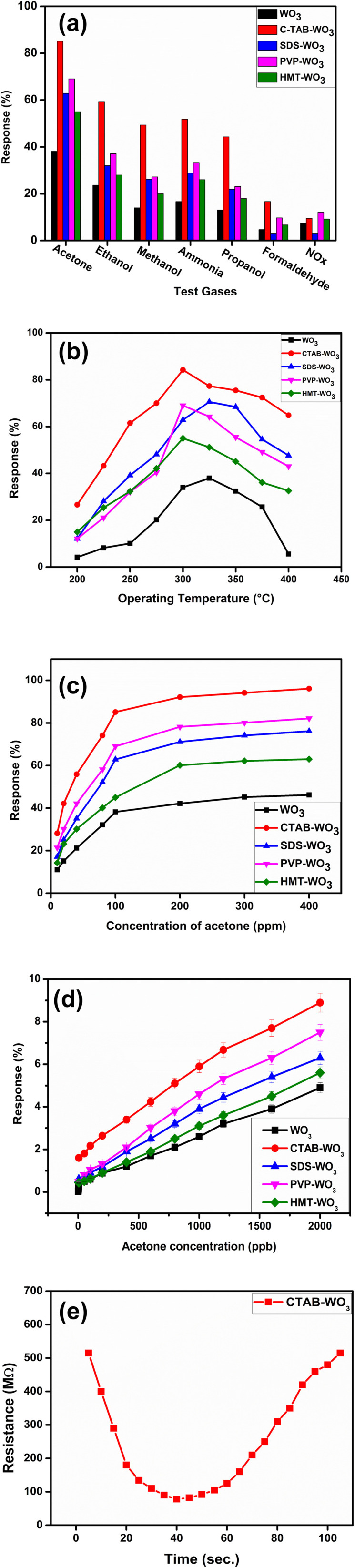
Gas sensing performance of pristine and surfactant-modified WO_3_: (a) selectivity, (b) operating temperature, (c) acetone concentration variation, (d) acetone sensing performance at ppb level and (e) response/recovery dynamics.

Fig. 11a shows the sensor response of each indidual WO_3_ material (without and with surfactant) towards various reducing and oxidizing vapors, including acetone, methanol, ethanol, propanol, formaldehyde, ammonia, and NO_*x*_ at 100 ppm test gases with an operating temperature of 300 °C. Amongst all the sensors, only CTAB–WO_3_ sensor shows the highest response towards acetone, ethanol, methanol, ammonia, propanol and formaldehyde indicating responses of 84.84%, 59.37%, 49.36%, 51.85%, 44.28% and 16.66%, respectively. CTAB-assisted WO_3_ monoclinic crystal structure exhibits the highest response due to enhanced surface area, and oxygen vacancies compared with pristine WO_3_ and another surfactant.

#### 3.1.3 Operating temperature

The sensing response *vs.* operating temperature of the developed sensors are shown in Fig. 11b. This investigation included a temperature range covering from 200 °C to 400 °C, where the sensors showed – (i) the increased response, (ii) attain a peak value, and (iii) eventually drops with further increasing operating temperature. The sample shows a maximum response corresponding to a particular operating temperature. Pristine WO_3_, CTAB, SDS, PVP, and HMT–WO_3_ exhibited response of 34.04%, 84.84%, 62.88%, 69.02%, and 55.01% towards 100 ppm acetone at 300 °C operating temperature. This ensures the high sensitivity and rapid sensor response, making CTAB–WO_3_ superior at its specific operating temperature.

#### 3.1.4 Concentration variation

At an optimized operating temperature of 300 °C, the effect of acetone concentration on gas response was evaluated for pristine WO_3_ and surfactant-assisted WO_3_ samples (Fig. 11c). The results show a linear relationship between gas response and acetone concentration up to approximately 200 ppm, beyond which saturation occurs. The sensor's sensitivity is governed by removing adsorbed oxygen molecules through reactions with acetone, releasing electrons. Fewer gas molecules interact with the sensor surface at lower gas concentrations, limiting surface reactions. As the gas concentration increases, the number of surface reactions rises, leading to a higher response. However, once surface coverage approaches saturation, additional gas molecules have a diminished effect, resulting in a slower increase in response. Higher surface area, increased oxygen vacancies, and optimized charge transfer factors allow the CTAB–WO_3_ monoclinic crystal structure to maintain superior sensitivity, a strong linear response, and a higher maximum response before reaching saturation.

The Fig. 11d illustrates the low ppb error bar of the gas sensing performance of WO_3_ (tungsten trioxide) and with different surfactants (CTAB, SDS, PVP, and HMT) response towards acetone vapors with various concentrations in (ppb) level. When acetone vapor concentration of 1000 ppb is injected onto the surface of the sensor, the response of pristine WO_3_ and surfactant-assisted WO_3_ (modified with CTAB, SDS, PVP, and HMT) was 2.6%, 5.9%, 3.9%, 4.6% and 3.1%, respectively. Pristine WO_3_ exhibited the lowest response of 0.42% at 5 ppb acetone at 300 °C operating temperature. In contrast, CTAB-modified WO_3_ demonstrated the highest response, exceeding 1.6% for 5 ppb concentration. Overall, incorporating surfactants substantially enhances the gas-sensing performance of WO_3_, with CTAB-modified WO_3_ showing the most promising sensitivity for acetone vapor detection.

#### 3.1.5 Response/recovery dynamics

Fig. 11e depicts the dynamic response and recovery behaviour of a CTAB–WO_3_ based gas sensor when exposed to acetone vapor. Initially, the sensor exhibits a high baseline resistance of approximately 515 MΩ in air atmosphere. When acetone vapour is exposed to the sensor, it declines sharp in resistance with minimum value of about 78 MΩ at 40 s. This sharp decrease in the resistance indicates the sensor's effective response to acetone, driven by the adsorption of acetone molecules onto the surface of the sensor. This leads to an increase in charge carrier density, thereby reducing the overall resistance. The sensor's response is measured as 84.84% of a given formula (4). The acetone exposure on the sensor enters a recovery phase, where the resistance gradually increases as the adsorbed acetone molecules desorb from the sensor surface. This desorption process allows the sensor to regain its initial resistance, achieving substantial recovery within 60 s. The observed response and recovery times highlight the sensor's potential for real-time acetone detection, with a rapid response to the target gas and efficient recovery, which are critical parameters for practical gas sensing.

**Fig. 12:**
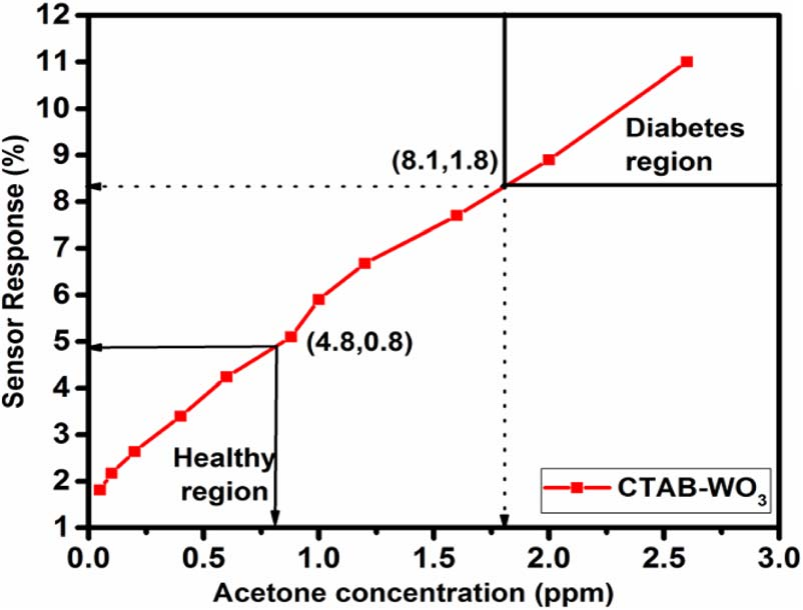
Acetone sensing response of CTAB–WO_3_ sensor: differentiating healthy and diabetes regions.

For a healthy person, acetone in breath averages less than 0.8 ppm, and higher than 1.8 ppm in a diabetic person.^24^ The following graph Fig. 12 shows the CTAB–WO_3_ sensor with varying concentrations of acetone, expressed in parts per million (ppm). The percentage response measures the sensor's performance, which increases as the acetone concentration rises. The graph is distinctly divided into two regions: the “Healthy region” at lower acetone levels and the “Diabetes region” at higher levels. A critical transition occurs at approximately 0.8 ppm, where the sensor response is nearly 4.8%, signifying the boundary between the healthy and diabetic states. As the acetone concentration continues to increase, the sensor's response also rises, with a notable value of nearly 8.1% at around 1.8 ppm, a level associated with diabetes. This linear response across the concentration range demonstrates the CTAB–WO_3_ sensor's ability to effectively differentiate between healthy and diabetic acetone levels, making it a promising tool for non-invasive diabetes monitoring.^25,26^

**Fig. 13:**
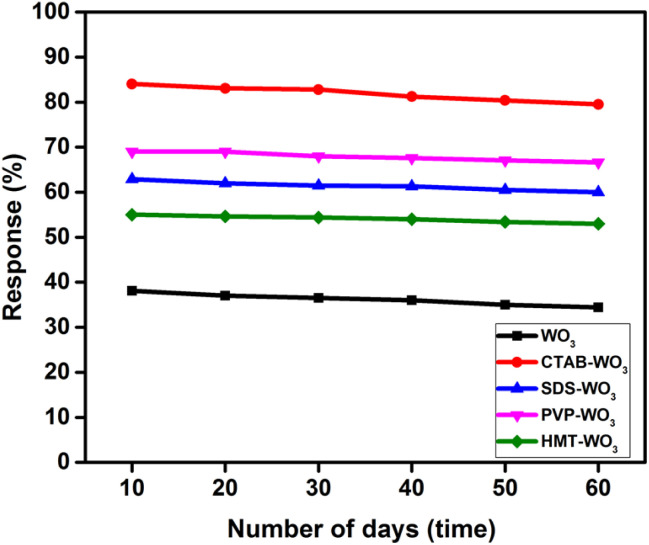
Long-term stability of sensors response over 60 days.

The stability and reproducibility of the all sensors were assessed, as illustrated in Fig. 13. The evaluations were conducted at an optimized operating temperature of 300 °C, with the sensor's response to a 100 ppm acetone concentration being measured. The sensor was tested initially and then periodically every ten days over two months. Results indicate that the pure WO_3_, CTAB–WO_3_, SDS–WO_3_, PVP–WO_3_, and HMT–WO_3_ sensor maintains approximately 35%, 81%, 61%, 67% and 54% of its initial response after this duration, demonstrating significant stability and reproducibility.

### 3.2 Photocatalytic degradation of methylene blue

The photocatalytic degradation of methylene blue (MB) involves a sequence of reactions driven by natural sunlight energy. Upon illumination with light of sufficient energy, the photocatalyst, such as CTAB–WO_3_, absorbs photons, which promote electrons from the valence band to the conduction band, creating electron–hole pairs. These charge carriers migrate to the surface of the catalyst, where they initiate redox reactions. The electrons interact with oxygen molecules, producing superoxide radicals, while the holes oxidize water or hydroxide ions to form hydroxyl radicals. These reactive species break down methylene blue into intermediate compounds, which are subsequently degraded into harmless products such as carbon dioxide and water. This process demonstrates the effectiveness of photocatalysis as a sustainable method for the removal of organic pollutants from aqueous solutions. The photocatalytic activity of pristine and surfactant-assisted WO_3_ samples was evaluated *via* the degradation of methylene blue under natural sunlight irradiation, as detailed in the SI (SI-III).

Further, the developed samples were evaluated for photocatalytic dye degradation by monitoring the degradation of methylene blue (MB) under natural sunlight. The degradation process was monitored over time by measuring changes in MB concentration using UV-Vis spectroscopy. The absorbance of effluents within the range of visible light (*λ* = 350–750 nm) irradiated under sunlight exposure with the interval of every 30 min, observing variations in the concentration of MB dye, particularly at its characteristic absorption peak of 665 nm, were carried out. The dye degradation efficiency was calculated using the following formula:

10 *η* (%) = ((*C*_i_ − *C*_f_))/*C*_i_ × 100

where, *η* (%) represents the degradation efficiency, *C*_i_ is the dye concentration before irradiation and *C*_f_ is the dye concentration after irradiation of sunlight.

**Fig. 14:**
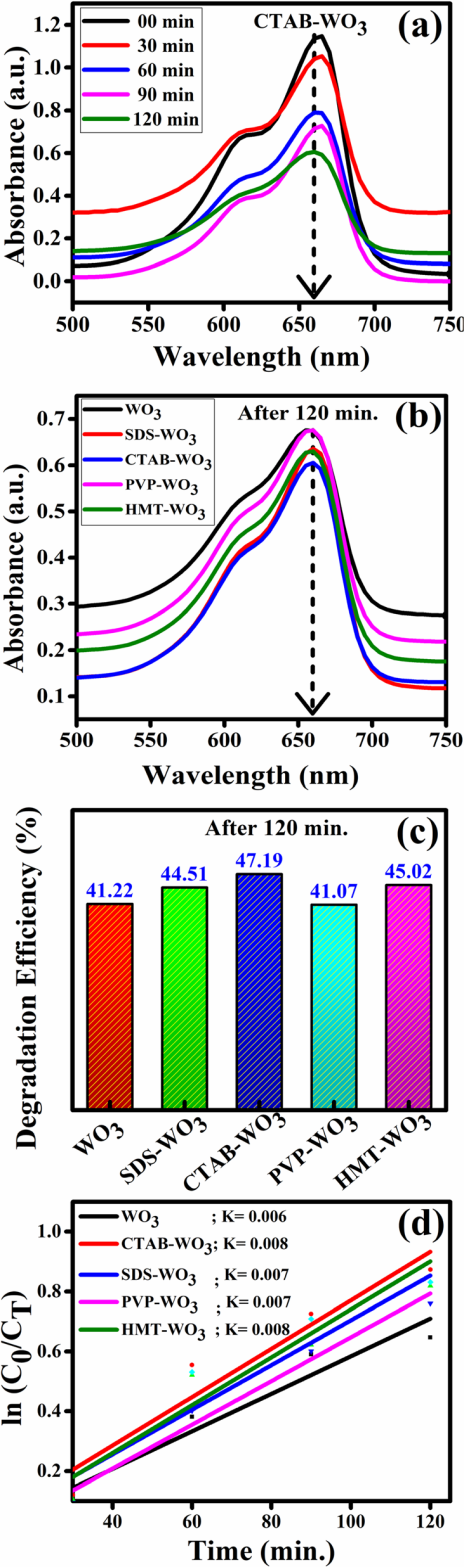
Photocatalytic activity of surfactant-assisted WO_3_: (a and b) absorbance spectra, (c) degradation efficiency and (d) pseudo-first-order kinetics.

There is a significant enhancement in dye degradation observed in the pristine WO_3_ after being treated with different surfactants as shown in Fig. 14a–c.

MB dye-degradation efficiencies for two hours of solar irradiation of pristine WO_3_ and various surfactant-assisted WO_3_ depends on surface area, charge transfer, and active sites on the surface of material. CTAB–WO_3_ showed the highest efficiency (47.19%) due to enhanced surface area and better charge separation. HMT–WO_3_ (45.02%) and SDS–WO_3_ (44.51%) performed moderately well, while pristine WO_3_ (41.22%) and PVP–WO_3_ (41.07%) had the lowest efficiency due to fewer active sites. CTAB–WO_3_ proved more effective for photocatalytic activity. These results are compared using pseudo first order model,

11 ln(*C*_0_/*C*_*t*_) = *K*_APP_*t*

where *C*_0_ = initial absorbance (at ∼664 nm), *C*_*t*_ = absorbance at time *t* (30 min 60 min 90 min and 120 min), *K*_APP_ = apparent rate constant, *t* = irradiation time.

From Fig. 14d, it is confirmed that CTAB–WO_3_ exhibits higher rate constant than pristine WO_3_, and follows pseudo-first-order kinetics reasonably well. This kinetic analysis demonstrates that the MB degradation by WO_3_ and surfactant-assisted WO_3_ samples fits well to a pseudo-first-order model. The linearity of the plots and calculated rate constants further confirms enhanced degradation performance in the presence of surfactants, especially CTAB. This result shows that the surfactants play a crucial role in increasing the surface area of the original material, thereby boosting its photocatalytic activity.

Comparative table that highlight the key properties of the WO_3_ based material for gas sensing and photocatalysis study *versus* those reported in recent literature as shown in Table 3.

**Table 3:** Comparison table of WO_3_ nanostructures developed *via* different routes and their obtained properties

Material	Method	BET surface area (m^2^ g^−1^)	Pore diameter (nm)	Gas response (100 ppm acetone)	Photocatalytic efficiency (MB, %)	Reference
WO_3_ nanoplate	Hydrothermal method	4.865	11.81	50.00%	—	27
Macroporous WO_3_	Hydrothermal method	0.0055	19.74	23.08%	—	23
Semi-cubic WO_3_	Hydrothermal method	25.47	18.49	76.30%	—	16
WO_3_-500	Calcination assisted template method	8.39	—	5 ppm, 8	—	28
WO_3_ nanoflakes	Green synthesis	13	19.30	—	90%	29
WO_3_ nanoparticles	Hydrothermal method	15.48		10 ppm, 84%	—	30
WO_3_ nanofibers	Electrospinning method	11.40	7.95	11	—	31
CTAB–WO_3_ (this work)	Hydrothermal method	4.22	31.39	84.84%	47.19%	Present work

Overall physicochemical properties, gas sensing and photocatalytic performance of pristine WO_3_ and surfactant-assisted WO_3_ samples are shown in Table 4.

**Table 4:** Comprehensive performance table of developed WO_3_ with and without surfactants

Parameter	Pure WO_3_	CTAB–WO_3_	SDS–WO_3_	PVP–WO_3_	HMT–WO_3_
Crystallite size (nm)	22.4	**21.56**	22.27	23.64	23.25
Dislocation density (10^16^ lines per m^2^)	0.199	**0.215**	0.201	0.179	0.185
Microstrain (10^−2^ lines per m^4^)	0.74	**0.78**	0.75	0.72	0.73
FESEM morphology	Rectangular nanoplate	**Small porous nanoplate**	Faceted nanoplate	Larger nanoplate	Irregular nanoplate
Particle size (nm)	226	**156**	188	215	142
BET surface area (m^2^ g^−1^)	2.26	**4.22**	2.18	1.49	0.99
Pore diameter (nm)	38.14	**31.39**	34.54	29.98	53.55
Pore volume (cm^3^ g^−1^)	0.021	**0.033**	0.019	0.11	0.013
Selectivity (response at 100 ppm, 300 °C)	**Acetone: 34.04%**	**Acetone: 84.84%**	**Acetone: 62.88%**	**Acetone: 69.02%**	**Acetone: 55.01%**
	Methanol: 20.12%	**Methanol: 49.36%**	Methanol: 37.56%	Methanol: 42.33%	Methanol: 34.21%
	Ethanol: 24.56%	**Ethanol: 59.37%**	Ethanol: 45.23%	Ethanol: 48.12%	Ethanol: 39.67%
	Propanol: 15.87%	**Propanol: 44.28%**	Propanol: 32.12%	Propanol: 35.43%	Propanol: 29.87%
	NH_3_: 5.61%	**NH**_**3**_ **: 9.52%**	NH_3_: 8.31%	NH_3_: 7.52%	NH_3_: 6.72%
	NO_*x*_: 10.23%	**NO**_***x***_ **: 51.85%**	NO_*x*_: 41.45%	NO_*x*_: 46.87%	NO_*x*_: 38.94%
Optimal temperature (°C)	300	300	300	300	300
Response at optimal temp. (100 ppm acetone)	34.04%	84.84%	62.88%	69.02%	55.01%
Response time (s)	50	**35**	40	45	50
Recovery time (s)	80	**70**	75	78	85
Efficiency of decolonization (% MB)	41.22%	**47.19%**	44.51%	41.07%	45.02%

## 4. Summary and conclusion

The pristine WO_3_ and various surfactant assisted WO_3_ (CTAB, SDS, PVP and HMT) nanostructure are developed *via* a simple one-step hydrothermal route and systematically analyzed using XRD, FESEM-EDAX, TEM/HRTEM, XPS, UV-Vis, and BET measurements. Furthermore gas sensing and photocatalytic activity are studied. All synthesized samples exhibit monoclinic crystal structure with nanoplate-like morphology. Surfactant CTAB plays a major role, in exhibiting small crystallite size and high surface area of WO_3_ nanostructure, further indicating suitability for gas sensing and photocatalytic applications. The CTAB–WO_3_ sensor showcase 84.84% response towards acetone (100 ppm) at 300 °C operating temperature, with a quick response time of 35 s and a recovery time of 70 s. In contrast, SDS–WO_3_, PVP–WO_3_ and HMT–WO_3_ displayed relatively low surface area, ultimately leading to a moderate acetone response. Among all samples, CTAB–WO_3_ demonstrated superior selectivity and sensitivity towards acetone compared to methanol, ethanol, propanol, ammonia, and NO_*x*_. Additionally, CTAB–WO_3_ exhibited the highest efficiency for methylene blue decolourization (47.19%), highlighting its dual functionality in gas sensing and photocatalysis applications. The enhanced performance of WO_3_ is attributed to the incorporation of CTAB, which acts as a cationic surfactant, capping agent, and structure-directing agent during the synthesis process. This modification results in a significantly increased surface area and a markedly improved gas sensing response towards acetone. Additionally, the CTAB–WO_3_ nanostructures exhibit excellent photocatalytic activity for the degradation of methylene blue dye under natural sunlight.

## Conflicts of interest

The authors declare no competing interests.

## Supplementary Material

RA-015-D5RA02593A-s001

## Data Availability

The data supporting this article have been included as part of the SI. Supplementary information on characterization techniques used (SI-I), sample preparation for gas sensing (SI-II) and photocatalytic activity test (SI-III) is available. See DOI: https://doi.org/10.1039/d5ra02593a.
