# Supplementary material for: Surfactant-assisted tungsten oxide for enhanced acetone sensing and solar-driven photocatalysis: exploring the role of surfactants
Source: RSC Adv. 2025 Aug 5;15(34):27895–907. doi: 10.1039/d5ra02593a (PMC12322810; doi:10.1039/d5ra02593a)
Supplement: RA-015-D5RA02593A-s001 [file RA-015-D5RA02593A-s001.pdf]

Supplementary Information (SI) for RSC Advances.
This journal is © The Royal Society of Chemistry 2025

Supporting Information

Surfactant Assisted Tungsten Oxide for Enhanced Acetone Sensing and Solar Driven Photocatalysis: Exploring the Role of Surfactants

Abhijeet P. Patil,[a] Suraj S. Patil,[a,b] Mohaseen S. Tamboli,[c*] Shubhangi R. Damkale,[d] Digambar Y. Nadargi,[a,e*] Jyoti D. Nadargi,[f] Imtiaz S. Mulla,[g] and Sharad S. Suryavanshi[a*]

[a] School of Physical Sciences, Punyashlok Ahilyadevi Holkar Solapur University, Solapur - 413255, Maharashtra, India. Email-sssuryavanshi@rediffmail.com.

[b] Department of Physics, Yashavantrao Chavan Institute of Science, Satara-415001, India.

[c] Korea Institute of Energy Technology (KENTECH), 21 KENTECH-gil, Naju, Jeollanam-do, 58330, Republic of Korea. Email- tamboli.mohseen@gmail.com.

[d] Centre for Materials for Electronics Technology (C-MET), Off Pashan Road, Panchawati, Pune-411008, Maharashtra, India

[e] Centre for Materials for Electronics Technology, (C-MET), Thrissur -680581, Kerala, India. Email-digambar_nadargi@yahoo.co.in

[f] Department of Physics, Department of Physics, Santosh Bhimrao Patil College, Mandrup, Solapur-413221, India.

[g] Former Emeritus Scientist, (C-MET), National Chemical Laboratory, Pune-411008, India

E-mail: tamboli.mohseen@gmail.com, digambar_nadargi@yahoo.co.in,

sssuryavanshi@rediffmail.com

Supporting information I – Characterizations

Supporting information II - Sample preparation for gas sensing

Supporting information III - Photocatalytic activity test

1. Supporting information I – Characterizations:

Investigations of crystallinity and phase identification of both pristine WO_3 and various surfactant assisted WO_3 was carried out using X-ray diffraction (Model-Bruker D8 advanced X-ray diffractometer). The XRD data was collected at slow scan rate of 2o/minute in a wide range of Bragg's angle 2θ (10o< 2θ< 60o) with Cu-Kα radiation (λ =1.5406Å). The surface morphology was analysed using FE-SEM (Model 6306A JEOL-JSM, Japan) and TEM techniques (HITACHI HF 5000). To confirm the elements present in the sample as well as oxidation states of samples X-ray photoelectron spectroscopy (XPS) PHI 5000 VersaProbe (Ulvac-PHI) was used. Optical band gap of all sample finding use UV-visible spectroscopy (UV-2600i SHIMADZU). Analysis of surface area and pore volume in the sample use Brunauer–Emmett–Teller (BET) analyzer (BET, BELSORP-min II, Bel Japan, Inc. Japan). Gas sensing analysis of the developed material was carried out using indigenous 2L gas sensing chamber.

2. Supporting information II - Sample preparation for gas sensing:

For device fabrication, an appropriate quantity of pristine WO_3 and various surfactant assisted WO_3 powder was finely ground with a small amount of butyl carbitol acetate using an agate mortar to form a homogeneous slurry. The resulting slurry was uniformly deposited onto an alumina substrate via the screen-printing technique. The coated substrate was allowed to dry at room temperature for 30 minutes, followed by thermal treatment at 200°C for 2 h to eliminate organic binders. Finally, silver electrodes were applied to facilitate electrical measurements. The gas sensing measurements were conducted using a glass assembly equipped with a ceramic sample holder featuring two probes, where the sensor was positioned coaxially within a temperature-controlled furnace as shown in Fig. S1. The sensing performance was assessed by monitoring the resistance change of the sensor before and after exposure to the target gas.

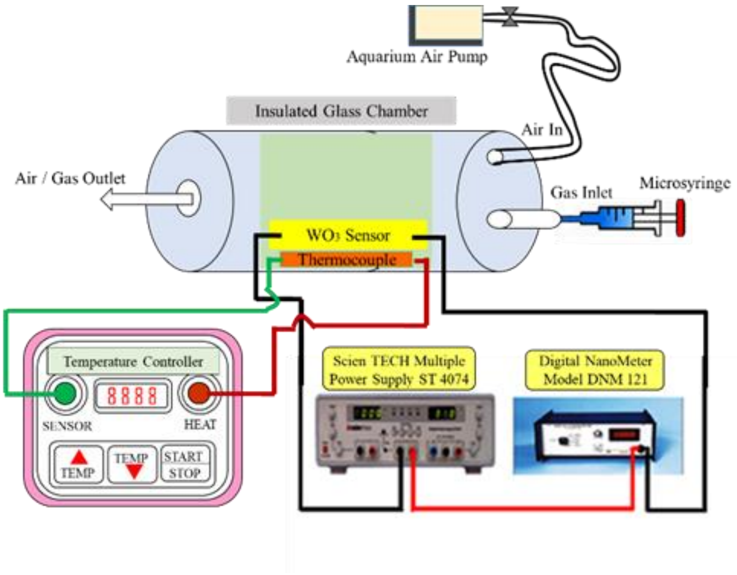

Figure S1: Schematic illustration of gas sensing unit.

3. Supporting information III - Photocatalytic activity test:

To study the photo-catalytic behaviour of pristine and surfactant assisted WO_3 samples, the photo-catalytic degradation of methylene blue dye was carried out under natural sun light irradiation. Initially, aqueous solution of methylene blue dye (5 mg/l) is prepared in 100 ml of distilled water. As prepared WO_3 sample (0.1 g) was dispersed in 100 ml of dye solution as a photo catalyst. The suspension was magnetically stirred for 60 min in the dark, before putting it under natural sunlight. The solution was finally irradiated under the natural sunlight with constant stirring. Methylene blue dye degradation was confirmed by the analysis of UV–Vis absorption spectra recorded at interval of every 30 min.

Photocatalytic mechanism:

When sunlight falls on the CTAB-WO_3 nanoplate, it absorbs photons because of its suitable bandgap of 2.77 eV. This energy excites electrons from the valence band to the conduction band, creating electron-hole pairs. These charge carriers migrate to the surface, where electrons reduce adsorbed oxygen molecules to form superoxide radicals ($\bullet O_2^-$),), and holes oxidize water or OH^- ions to produce hydroxyl radicals (•OH). These reactive species then attack and degrade methylene blue (MB) dye molecules present on the surface, breaking them down into harmless products like CO_2 and H_2O. Hence, WO_3 shows efficient sunlight-driven photocatalytic activity for MB dye degradation. This photocatalytic mechanism and fig.S_2 added in main manuscript.

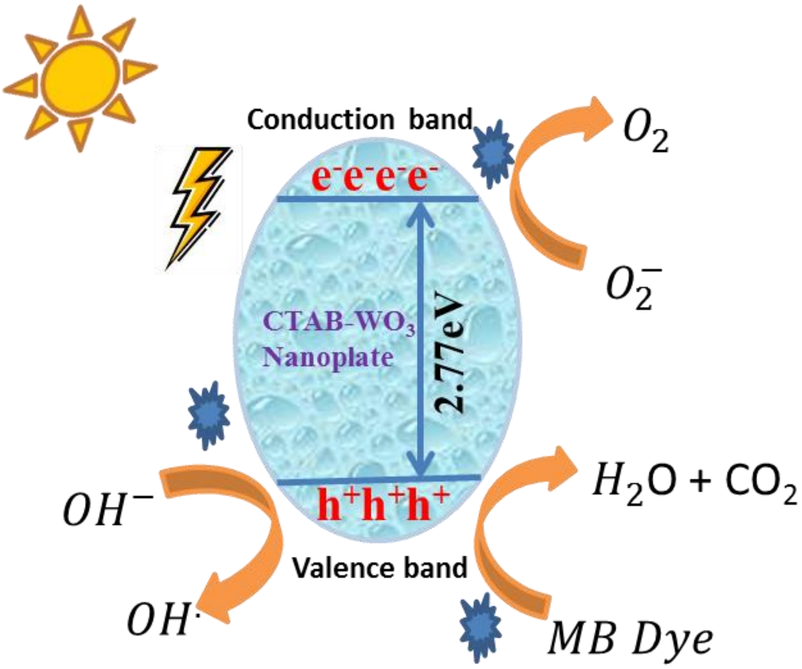

Fig. S3 The sunlight-driven photocatalytic mechanism of WO_3 nanoplate for the degradation of methylene blue (MB)
